# Routine Electronic Mother-Infant Data (REMInD): a proof-of-concept Data to Care study to support retention in maternal HIV treatment and infant HIV testing in Cape Town, South Africa

**DOI:** 10.21203/rs.3.rs-5626699/v1

**Published:** 2024-12-13

**Authors:** Tamsin K. Phillips, Yolanda Gomba, Pheposadi Mogoba, Florence Phelanyane, Kim Anderson, Benjamin H. Chi, Kate Clouse, Mary-Ann Davies, Jonathan Euvrard, Lucia Knight, Landon Myer, Elaine J. Abrams

**Affiliations:** University of Cape Town

**Keywords:** Data to Care, vertical HIV transmission prevention, retention, early infant diagnosis, South Africa

## Abstract

Data to Care (D2C) strategies – using routine data to facilitate identification and linkage back to care of people living with HIV who are not in care – have shown promise in high-income settings but received little attention in lower resourced or vertical HIV transmission prevention (VTP) contexts. In this proof-of-concept study, we monitored existing linked electronic medical records in near real-time to identify key gaps in postpartum VTP steps among 336 mothers living with HIV and their infants in Cape Town, South Africa (recruited March 2021 – April 2022). We attempted to confirm observed gaps through source data systems and telephonic tracing, and facilitated re-engagement in care where needed. There were 302 gaps observed in the routine data; 123 (41%) were false gaps and 179 (59%) were considered probable gaps (133 mother-infant pairs). Overall, 54 mothers (16%) did not link to HIV care within 12 weeks of delivery, 43 mothers (13%) linked to care but had a gap in ART dispensing by nine months postpartum, 25 infants (10%) did not have an HIV test around 10 weeks and 57 (17%) had no HIV test around 6 months of age. Only 100 of the probable gaps (56%) could be confirmed through telephonic tracing and, of those, only 47 were successfully re-linked to care. Mobility and clinic transfer, fear of stigma and employment-related challenges were commonly reported reasons for gaps in VTP steps. This study highlights that linked routine data sources linking mother-infant pairs across health facilities has the potential to streamline tracing efforts; however, implementation is challenging and, even when gaps are identified, re-engagement in care may be difficult. Further research is needed to combine D2C strategies with interventions addressing broader social and structural determinants of health, and to tailor D2C strategies to fit available resources and data sources in low-resource settings.

## Introduction

Despite substantial progress towards the elimination of vertical transmission of HIV and impressive declines in new HIV infections in children worldwide, important gaps remain in early infant diagnosis and continuity of maternal antiretroviral therapy (ART) postpartum [1]. In South Africa, HIV transmission in the breastfeeding period has overtaken perinatal transmission with > 50% of new child infections attributed to breastfeeding [1]. Postpartum maternal ART interruptions and incomplete infant HIV testing at recommended time points are common [2, 3].

Data to Care (D2C) is a collaborative public health strategy that supports the use of routine HIV surveillance data to facilitate the identification and linkage to care of people living with HIV who are not in care [4]. Experiences with D2C strategies have been predominantly focused on re-engagement of adults living with HIV in the United States (US), with encouraging evidence that employing D2C strategies within health departments can lead to improvements [5]. D2C programs in the US have highlighted the value of combining routine data sources to ensure a more real-world reflection of engagement in care and to assist with prioritizing scarce resources [6]; however, this approach has not been explored at scale in low- and middle-income countries (LMICs). To our knowledge, there has only been one published study of D2C strategies for people living with HIV in Africa: a pilot study in Mozambique that found when data from facility- and community-based services were combined to support children and adolescents living with HIV, improved retention and viral load testing uptake were observed [7].

D2C strategies may be particularly valuable to vertical HIV transmission prevention (VTP) programs. In South Africa and many other LMICs, mothers living with HIV and their infants move between locations of care to access VTP services over time [8]. Women may need to change clinic locations to receive ART and antenatal care during pregnancy, or they may be transferred out of integrated antenatal care and HIV services to routine ART clinics after delivery. These clinic transfers have been shown to present a risk for disengagement and poor outcomes [9, 10]. Routine transfers between clinics and individual geographic mobility also present a challenge for monitoring continuity of care, and ignoring transfers can result in large underestimates of retention [11, 12].

In the Western Cape (WC) province of South Africa, available public sector electronic health information systems are harmonized at the Provincial Health Data Centre (PHDC) to create an individual electronic medical record for patient care and health service management [13]. All people accessing public sector health services are allocated a unique identifier that allows linkage of health data across health facilities and information systems in the province. The PHDC was used extensively to support contact tracing and patient management during the COVID-19 pandemic [14, 15] and a strategy comparable to D2C has been used to support linkage between tertiary and primary care for patients diagnosed with tuberculosis [16]. In the Routine Electronic Mother-Infant Data (REMInD) study, we assessed a) the validity of PHDC data to identify infants with incomplete HIV testing and mothers with ART interruptions after delivery, and b) whether a proof-of-concept D2C strategy could be used to trace mother-infant pairs (MIPs) with gaps in VTP steps and facilitate re-engagement in care.

## Methods

### Local context

This study was centered around a large midwife obstetric unit (MOU) in Gugulethu, Cape Town. The MOU serves a wide catchment area with an estimated population of 400 000 people [17] and provides perinatal care, including HIV care, to about 5 000 women annually. The MOU is situated within a network of primary care clinics that provide basic antenatal care, routine baby and child health care, and HIV services. All primary healthcare services are provided free of charge in the public sector in South Africa.

During pregnancy, women start antenatal care at the MOU or a nearby primary healthcare clinic and then transfer to the MOU in the third trimester for continued care closer to delivery. Similarly, women already on ART are transferred into integrated antenatal and HIV care services at the MOU. Delivery takes place at the MOU or a nearby referral hospital if a caesarean section or more specialized care is required. Following delivery, mothers and infants have a visit at the MOU 7–10 days postpartum, at which mothers are dispensed 2–3 months of ART and transferred back to their nearest primary healthcare clinic for routine baby and HIV care. Babies receive HIV prophylaxis for 6–12 weeks, depending on level of risk, and are seen at primary healthcare clinics from 6 weeks postpartum for routine baby care including HIV testing. Care after delivery may be provided in an integrated model with mother and child attending the same clinic on the same day or, depending on the mother’s preference and available services, mother and child may access care on different days or at different clinics.

Robust provincial systems, coordinated at a substructure level, are in place for monitoring and tracing new infant HIV infections and to ensure linkage to treatment. However, tracing of gaps in routine ART or infant testing is limited to facility-specific missed appointment lists and patient tracing activities. When a mother or baby does not successfully transfer from the MOU back to primary care, or if they move to a different clinic postpartum, they will not be known to the receiving clinic as missing and would not appear on a missed appointment list until after they have attended the clinic and missed a subsequently scheduled visit.

### The REMInD study activities

**Figure 1:**
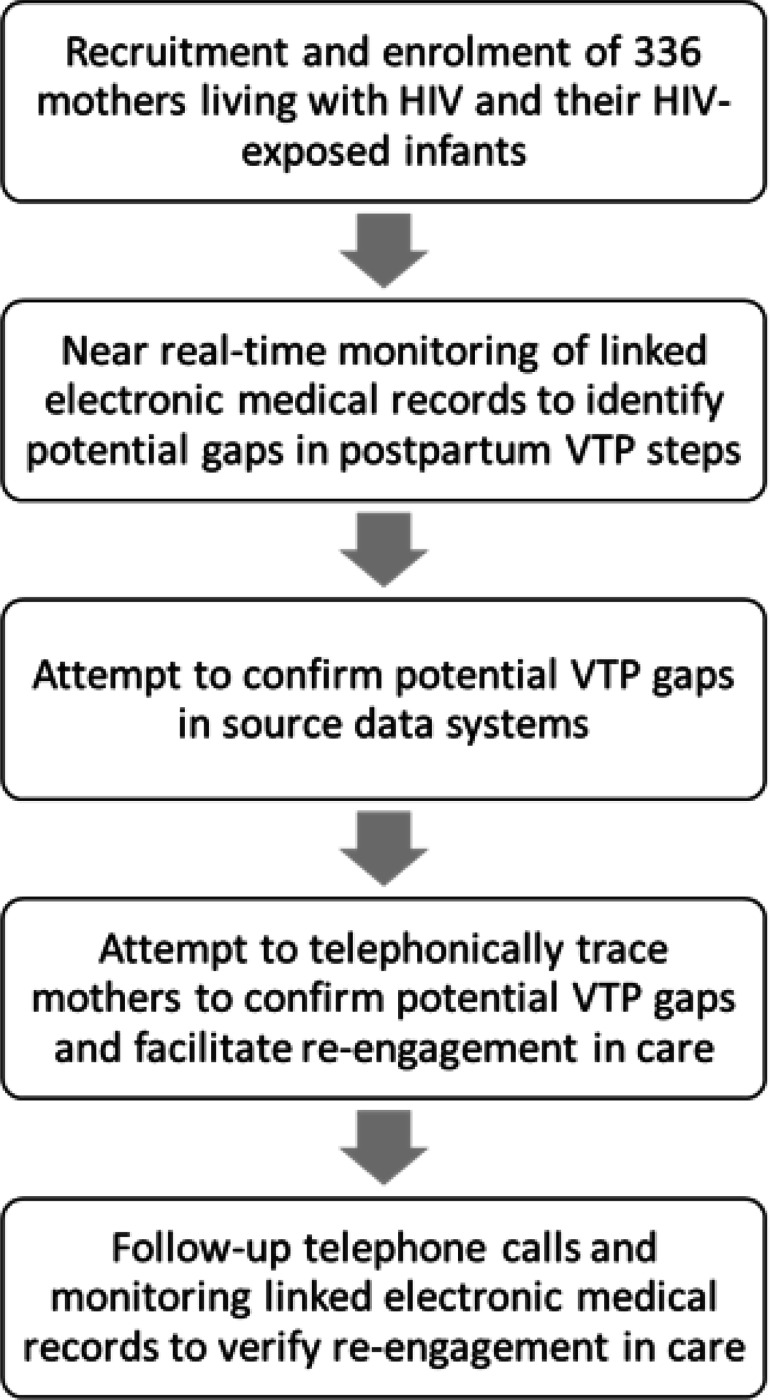
Summary of study activities. All activities in this proof-of-concept study were conducted by research staff (VTP – vertical HIV transmission prevention)

In a prospective cohort we 1) reviewed linked routine electronic medical records available in the PHDC in near real-time to observe gaps in key postpartum VTP steps, 2) attempted to confirm any observed gaps in source data systems, 3) attempted to telephonically contact participants to confirm care status and facilitate re-engagement in care for confirmed gaps, and 4) monitored successful re-engagement in care using PHDC data. Study activities are summarized in Fig. 1 and each component is described in more detail below.

## Study recruitment and enrolment

Pregnant and early postpartum women living with HIV who were attending antenatal care at the Gugulethu MOU between March 2021 and April 2022 were approached by a trained research fieldworker and screened for eligibility (age ≥ 18 years, in the third trimester or up to three weeks postpartum). Those who were eligible and willing to participate provided written informed consent, including consent to link to and monitor their and their infants’ electronic medical records, and to be traced by the study team should a gap in care be observed. At enrolment, women completed a comprehensive interviewer-administered questionnaire including socio-demographics and HIV treatment history. Contact details, unique health identifier and date of birth were recorded and stored separately from the questionnaire data to facilitate telephonic tracing and linkage to routine medical record data in the PHDC during study follow-up.

### Identifying gaps in postpartum VTP steps

Using the linked PHDC data, we actively monitored completion of key VTP steps (Table 1) in near real-time from enrolment through nine months postpartum. The selected steps are required by all mothers living with HIV and their HIV-exposed infants after transfer from the MOU to primary care [18]. Due to the required transfer from MOU to primary care, and potential geography mobility, these steps were considered those most likely to be overlooked through routine facility tracing activities for missed appointments.

Study investigators reviewed the PHDC web-based individual patient record (Single Patient Viewer) and existing patient-level maternity and HIV cascade reports every 2–4 weeks to confirm care completed as expected and identify potential gaps in VTP steps.

### Validation of potential gaps in VTP steps

Validation was attempted to identify any false gaps (Table 2) observed in PHDC data. Firstly, all missing infant HIV tests were cross-checked in the National Health Laboratory Services (NHLS) database to determine if any laboratory tests were completed but not incorporated into the PHDC. Secondly, gaps in ART dispensing observed in PHDC data were cross-checked in the source City of Cape Town health information system to confirm that data from the local government information system were successfully being incorporated into the PHDC. While the PHDC is administered by the WC Provincial Department of Health and Wellness, clinics in the City of Cape Town are administered by either the province or the City of Cape Town local government. Different data systems are in use and these are combined in the PHDC. No discrepancies were observed in the City of Cape Town data over a period of ten months and this validation process was discontinued in December 2021.

Where no evidence of completing the VTP step was found through the above checks, we initiated telephonic tracing of mothers to obtain additional information on mother and infant engagement in care and verify the gap observed in the PHDC data. Through this process we were able to ascertain instances where data were unavailable due to the mother or infant receiving care outside of the WC or data were missing after accessing care in the WC due to data capture or health identifier problems.

### Tracing and linkage back to care

In this proof-of-concept study, all tracing activities were conducted by research staff and were not linked to routine clinic or community-based services. Once a gap in VTP care was observed in the routine data by the research team, details were shared with the study field team within a week and tracing was initiated. Tracing telephone calls and linkage activities were conducted by a trained research fieldworker at the level of a lay community health worker. Telephone contact attempts were made using the contact details provided at enrolment for mothers and their alternative contacts. When needed, additional contact information was obtained from routine medical records. At least three telephone contact attempts were made on different days of the week and at different times of day. When a mother was successfully contacted, details of mother and infant care completed and any missed VTP steps were recorded. If a VTP gap was confirmed, the fieldworker explored reasons for the gap (open-ended questions) and counselled the participant to encourage and facilitate relinkage to care. Mothers and infants were provided with referral letters to their preferred clinic to continue care. Follow-up calls were made to determine whether they had managed to reconnect to care or if additional assistance was required. Re-engagement in care was also monitored using PHDC data.

## Data analysis

To assess the validity of using existing PHDC data to identify infants with incomplete HIV testing and mothers with ART interruptions after delivery, we categorized all observed gaps in VTP steps as false or probable VTP gaps following validation and tracing (Table 2).

We described gaps in VTP steps using frequencies and proportions and described the proportion of infants diagnosed with HIV through nine months with an exact 95% confidence interval. We also explored the overlap of gaps in VTP steps for mothers and their infants. In the case of a maternal or infant death in the study period, the mother or infant was still included in the denominator and counted as having no gap in care after the time of death.

To assess whether a D2C strategy could be used to trace MIPs with gaps in VTP steps and facilitate re-engagement in care, we described the proportion of probable VTP gaps where we successfully contacted the mother telephonically, and the proportion of VTP gaps with telephonic contact that were successfully closed by linking the mother or infant back into care by the end of the nine-month follow-up period. Open-ended reasons for gaps in care collected during telephonic tracing were thematically coded into broad categories.

We compared the enrolment characteristics of mothers with one, two or more, or no probable gaps in VTP steps (confirmed and unconfirmed gaps combined). In supplementary analyses we also compared characteristics of mothers with gaps in each of the included steps.

### Ethics

This study was approved by the University of Cape Town Human Research Ethics Committee (HREC reference 513/2020). Participants with psychosocial concerns identified during the enrolment interviews or during tracing activities were referred to local support services at the Gugulethu Community Health Centre or nearby Non-Governmental Organizations for counselling and further assistance.

## Results

A total of 336 mothers were enrolled: 201 (60%) during the third trimester of pregnancy and 135 (40%) early postpartum (Table 3). Six mothers did not complete the enrolment questionnaire but were included in all other analyses. The median maternal age at delivery was 32 years (IQR 28–36, 14% were < 25 years old). Most of the cohort (85%) had started ART before the incident pregnancy (median six years since first ART initiation, interquartile range [IQR] 4–9 years) and 40% of these mothers reported that they had interrupted ART at least once before the pregnancy.

There were five infant deaths and no pregnancy losses or maternal deaths recorded during the study period. By nine months postpartum, five of 336 infants had been diagnosed with HIV (1.5% transmission; 95% CI 0.5–3.5; details in Supplemental Table 1). All five infants started ART immediately and remained in HIV care throughout the study period.

## Completion of VTP steps after delivery through nine months postpartum

**Figure 2:**
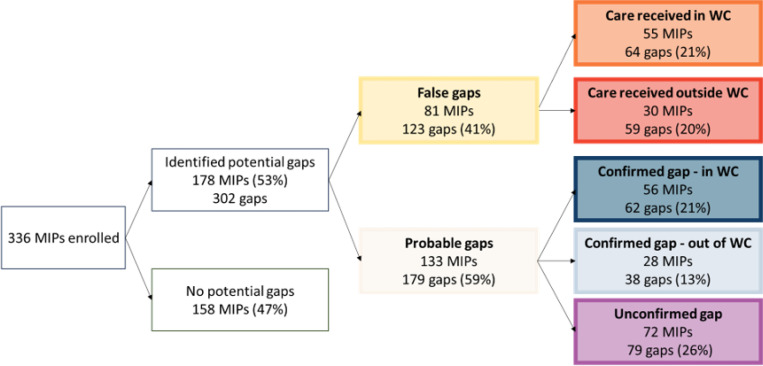
Classification of potential postpartum gaps identified in the Western Cape (WC) Provincial Health Data Centre among 336 mother infant pairs (MIPs).

**Figure 3:**
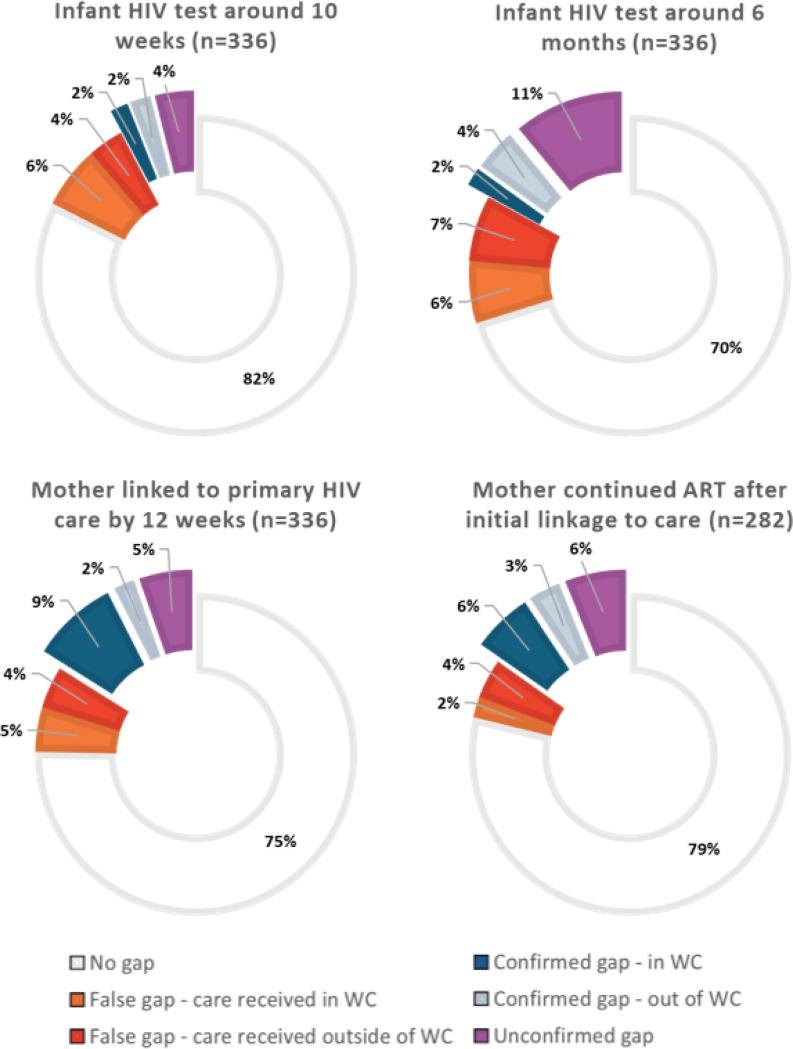
Classification of observed gaps in vertical transmission prevention steps identified in the Western Cape (WC) Provincial Health Data Centre among 336 mother infant pairs.

Overall, 158 MIPs (47%) had no observed gaps in VTP steps in the PHDC data. These MIPs had complete infant HIV testing at around 10 weeks and six months postpartum, the mother linked to care postpartum and had no gaps in ART dispensing up to nine months postpartum. There were 302 observed gaps in VTP steps in the remaining 178 MIPs when using PHDC data alone (Fig. 2). Through validation of source data and telephone calls with participants, 123 gaps (41%) were found to be false: 64 (52% of false gaps) were determined to be due to missing PHDC data after care was received in the WC, and for 59 (48% of false gaps) care was accessed outside of the WC so the data were unavailable in the PHDC. The remaining 179 gaps (59%) were classified as probable: 100 (56% of probable gaps) were confirmed through telephonic contact and 79 (44% of probable gaps) were unconfirmed. The breakdown of gaps for each VTP step is shown in Fig. 3. Mothers with any (n = 133) versus no (n = 203) probable gaps had similar characteristics except for age and ART history prior to the pregnancy (Table 3). Mothers with any probable gap were slightly younger (16% versus 9% under 25 years, p = 0.023) and more likely to be ART-experienced with a history of a previous interruption before the pregnancy (49% versus 24%, p < 0.001), compared to mothers with no probable gaps in VTP steps. Among the 133 MIPs with any probable gap, 93 (70%) experienced only one gap, 34 (26%) experienced two gaps and six (5%) experienced three gaps; differences appeared to be heightened among mothers with two or more probable gaps (26% under 25 years and 63% ART-experienced with history of a previous interruption) compared to those with only one probably gap (13% under 25 years and 43% ART-experienced with history of a previous interruption). Characteristics of mothers with gaps in each of the key VTP step are presented in Supplemental Table 2.

## Data feedback loops

Throughout the study, investigators provided feedback on data errors to the PHDC to facilitate improvement over time. Among the 64 false gaps following care received in the WC, 42 (66%) were due to infant HIV tests not successfully importing from the NHLS into the PHDC. A large data import change was made in January 2022. The proportion of infants with missing HIV tests due to data errors declined from 8.2% at 10 weeks (18/219) and 11.3% at 6 months (15/133) among infants with tests expected prior to February 2022, to 2.6% (3/117) and 2.9% (6/203) at 10 weeks and 6 months, respectively, among infants with HIV tests expected from February 2022 onwards. Other reasons for false gaps among participants accessing care in the WC included participants having multiple health identifiers that were not linked in the PHDC system (duplicate health identifiers were identified for 14 mothers), incorrect health identifier capture and, rarely, ART dispensing data not reflecting in the linked PHDC data.

### Success of D2C strategy to close gaps in VTP steps between delivery and nine months postpartum

The combined confirmed and unconfirmed gaps in VTP steps following validation totaled 179 probable gaps from 133 MIPs (40% of 336) (Table 4). Among 336 enrolled mothers, 54 (16%) did not link to HIV care within 12 weeks of delivery and 43 (13%) linked to care after delivery but subsequently had a gap in ART dispensing by nine months postpartum. Combined, 29% of all mothers had a gap in ART. Among 336 infants, 25 (10%) and 57 (17%) did not have an HIV test at around 10 weeks or 6 months of age, respectively; 23% of all infants missed either HIV test.

Only 100 of 179 mothers (56%) with probable gaps were successfully contacted telephonically and confirmed (Table 4). Of the 100 successfully traced, 47 gaps (47%) were subsequently closed with evidence of the mother or infant successfully relinking to care. A higher proportion of successful re-linkage to care was observed for the 10-week infant HIV test (83% of those contacted linked to care) and later postpartum ART gaps (66% of those contacted linked to care), compared to the 6-month infant HIV test (36% of those contacted linked to care) and mother linking to care after delivery (27% of those contacted linked to care). Among the 79 unconfirmed gaps, the main challenge was calls not being answered or going directly to voicemail (n = 53) and some were answered but reported to be incorrect numbers (n = 5). Some calls were answered by the participant who was unable to speak freely due to privacy concerns (n = 6). Four gaps were not traced as there was evidence of return to care prior to tracing, and for 12 gaps the reason for failed tracing was not recorded.

Reasons for not connecting to care were provided by mothers for 63 gaps (63% of the 100 successfully traced, Table 4). The most common reasons provided were related to geographic mobility and clinic transfer documentation challenges both within and outside of the WC. This included being turned away for not having documentation of treatment history, difficulty getting a transfer letter after moving out of the WC, and fear of not being served if they presented without transfer documentation. Other common reasons were fear of stigma or being scolded at the clinic and employment-related challenges.

## Overlap of mother and infant gaps in VTP steps

Little overlap was observed between gaps in VTP steps among mothers and their infants (Table 5). By 12 weeks postpartum, there were 53 MIPs (16%) where the mother had not linked to HIV care postpartum even though the baby had received a 10-week HIV test, and there were 10 MIPs (3%) where the baby had not completed a 10-week test, but the mother had linked to HIV care postpartum. By nine months postpartum, there were 41 MIPs (12%) where the mother was not accessing HIV care, but the baby had received a six-month HIV test, and there were 53 MIPs (16%) where the baby had no six-month test while the mother was engaged in HIV care.

### Discussion

In this study, we successfully used existing electronic medical record data (linked across data sources and public sector clinics) to screen out MIPs who had successfully completed key VTP steps within the WC, with close to 50% of the cohort completing all steps and requiring no further action. Lack of linked health data from outside the WC along with data import challenges and duplicate health identifiers in the WC resulted in some misclassification of gaps. Tracing MIPs with gaps in VTP steps and facilitating re-engagement in care in this low-resource setting in Cape Town, South Africa, proved challenging. Reassuringly, we observed low HIV transmission through nine months postpartum; however, we observed an ongoing need to strengthen postpartum ART continuity and infant HIV test completion. Our findings highlight the potential value and important challenges implementing a D2C strategy to identify and trace MIPs with gaps in VTP steps in this setting.

These results confirm the value of combining routine HIV data sources and using unique patient identifiers to link across health facilities, to obtain a more realistic view of engagement in HIV care and thus support more efficient patient tracing [6]. Geographic mobility in this study contributed both to misclassification of gaps and to reasons for gaps in VTP steps. In settings such as ours where mobility is common, improved health service adaptability is needed to ensure both patients’ continued engagement in care and continued provision of services despite changes in care location. Strategies to facilitate seamless clinic transfers are also urgently needed as this remains a substantial barrier, despite guidelines stating that people cannot be turned away from HIV care without transfer documentation [20]. While routine electronic data sources are not without challenges [21–24], the combination of multiple data sources linked across facilities allowed us to minimize missing data due to mobility, thereby minimizing the number of people misclassified as out of care. Harmonized data from multiple sources may not be available in all LMIC settings, or even HICs; however, it is still possible to implement similar strategies. A study implementing a D2C strategy in Mozambique, for example, held regular joint data reviews to compare and share facility and community data to identify children and adolescents who were out of HIV care [7]. Similarly, the CoRECT trial in the US held monthly meetings to consolidate lists of people out of care based on laboratory surveillance data and clinic appointment data [25, 26]. In both studies, joint reviews created an opportunity for collaboration between partners and for decisions to be made around appropriate next steps and support interventions. In Mozambique, such collaboration meant support services and linkage interventions could be moved from facilities to community-based services, allowing for greater geographic coverage and accommodation of mobility and movement between clinics [7].

Despite the value of a D2C approach, further research is needed to establish a robust evidence-base for D2C strategies in LMIC settings. Our study makes it clear that identifying who has had a gap in VTP care is an essential first step; however, that alone will not ensure continuity of care. Contacting mothers and facilitating linkage back to care was very challenging even in our research context with a dedicated field team. D2C strategies need to combine the strengths of routine HIV surveillance data with behavioral interventions to optimize relinkage and ongoing retention. All women in our study provided written informed consent to review their medical records and to trace them, yet telephonic tracing was only successful for 56% of MIPs with probable gaps. Among mothers who were contacted in our cohort, fear of stigma or being scolded at the clinic were among the primary reasons given for not connecting to care and this may also have influenced tracing success. Mutual patient-health service trust needs to be established to optimize the reach and impact of D2C strategies and in turn optimize service delivery and health outcomes. Consideration is also needed to ensure patients are aware of how their routine health data may be used and mechanisms to obtain consent for data use in routine health settings are essential [27].

It is well documented that barriers to engagement in HIV care are often related to broader social and structural factors not directly linked to HIV [28]. Mothers living with HIV, including those who interrupt HIV care postpartum or who do not complete HIV testing visits with their infants, are often living in unstable circumstances and need social support services in addition to HIV care [29, 30]. Although integration of care for MIPs is strongly recommended in the national ART guidelines [20], implementation remains complex [31]. It is not uncommon for infants in our setting to live apart from their mothers, with other primary caregivers [32], and after delivery women may not be identified as postpartum in routine HIV services. Challenges with mobility and clinic transfers in this cohort, as well as our finding of missed opportunities where mother and infant gaps in care do not overlap, may reflect some level of housing and economic instability during this vulnerable early postpartum period. Patients who received a D2C intervention in the US reported how the program helped to identify needs for social assistance as well as assisting with difficulties navigating HIV care [33]. Similarly, US providers felt that identifying and addressing social and structural barriers was often essential to enable re-engagement in HIV care and was a critical function of the D2C team [34]. Tracing efforts therefore need to be integrated with community-based services and incorporate links to required social support. It is also important to note that, while targeted tracing such as through D2C strategies can be useful to ensure short-term completion of care, there is a clear and urgent need for broader structural interventions to reduce the overall vulnerability in this population. A randomized D2C trial in the US found that although the D2C approach improved initial re-engagement in HIV care, it did not impact durable viral suppression [25]. In the context of large-scale investments made by international and national donors to improve data systems—alongside shrinking health budgets and growing program need—a D2C strategy should be one piece of a multicomponent approach to efficiently ensure completion of VTP steps, while also addressing broader social and structural barriers to improve health for all vulnerable mothers and their infants.

This work should be interpreted with the following limitations in mind. First, while the routine electronic medical record data in the WC links numerous data sources and uses a unique patient identifier to link individual data between facilities, this does not extend outside of the WC or to private sector health services. In previous research in this setting, we have documented mobility outside of the WC in 9% of mothers in the first two years postpartum [11]. While this is a limitation of the data, having these individuals on the list of patients out of care is a potentially important opportunity to ensure continuity of care. Our results highlight that connecting to infant HIV testing services and continued ART when traveling outside of the WC is a substantial challenge and additional support and improved data systems are necessary to facilitate and track interprovincial transfers. Second, this study recruited women accessing antenatal care at a single clinic in Cape Town, the D2C strategy was implemented by a research team and the study made use of a unique harmonized data system in the WC. While this limits the generalizability of the findings, the lessons learnt from this study are likely to apply to many high-HIV-burden settings on the continent where mobility is common and completion of VTP steps remains a challenge. There are continued efforts to implement electronic health records in many LMICs and electronic health record systems do exist in other African countries. The data sources incorporated in the WC electronic health data are largely available in the rest of South Africa and, along with a unique health identifier, mean that D2C strategies such as that presented in this study should be possible. Further research is needed to understand adaptations needed to incorporate tracing activities into routine health services and how best to tailor implementation strategies to specific contexts and their available data systems and resources. Finally, we observed low rates of infant HIV transmission in this study; however, follow-up was limited to six months and breastfeeding status is not recorded in the routine data, so we were unable to ascertain final infant HIV outcomes. The short follow-up duration also means we do not know the longer-term patterns of maternal HIV care engagement and we did not measure maternal viral load which will be an important indicator for future studies. HIV transmission through breastfeeding is an important contributor to new infant HIV infections and the incomplete infant HIV testing and maternal retention observed in this cohort highlights the need for ongoing investment in interventions to support postpartum retention in care.

In conclusion, in this proof-of-concept study of a D2C strategy to strengthen completion of VTP steps in South Africa, linked electronic data provided a more realistic view of VTP completion and allowed for targeted tracing of MIPs with probable gaps. Strategies that enhance trust between patients and health services, and that improve our ability to connect with people who are out of care, are urgently needed. Future research should consider adaptations needed to embed D2C strategies into routine health services in LMIC settings, and how to incorporate the strengths of D2C with links to community-based services and structural interventions that speak to the broader needs of women living with HIV and their children.

## Supplementary Material

Supplement 1

## Figures and Tables

**Table 1:** Key steps in vertical HIV transmission prevention (VTP) examined in the REMInD study

Name	Definition^a^	Existing routine data indicator
Infant HIV testing around 10 weeks	Evidence of an HIV polymerase chain reaction (PCR) test between 6 and 16 weeks of age	Yes
Infant HIV testing around 6 months	Evidence of an HIV PCR test between after 16 weeks and up to 36 weeks of age	Yes
Mother linked to primary HIV care after delivery	Evidence of ART dispensed at a primary care clinic after delivery and up to 12 weeks postpartum	No
Mother continued ART after initial linkage to care	Following linkage to primary care, no gap of > 12 weeks between ART dispensing dates	No
Infant with positive HIV PCR initiated and retained on ART	Evidence of ART initiation and continued dispensing of ART following infant HIV diagnosis	Yes

a Windows used in these definitions are based on the South African National Indicator Dataset [19]

**Table 2:** Categorization of gaps in vertical transmission prevention steps observed in the routine data following validation and patient tracing attempts.

Gap category	Description
**False gaps in VTP steps**	**All false gaps observed in the routine data due to data errors and unlinked data as defined below**
Care received in WC	*Care was received at a public sector clinic in the WC, but evidence of care access was not visible in the linked routine data. For example, an infant received an HIV test, but the test was not imported into the linked WC data*.
Care received outside the WC	*Participants contacted telephonically and reported that care was received outside of the WC. For example, a mother reported receiving ART from a clinic in another province. Data from clinics outside the WC is not included in the linked WC data.*
**Probable gaps in VTP steps**	**All confirmed and unconfirmed VTP gaps as defined below**
Confirmed gap in VTP step	*No evidence of accessing care could be found in the linked routine data or available source data. Participants contacted telephonically and confirmed that they had not accessed care and there was a gap in VTP steps.*
Unconfirmed gap in VTP step	*No evidence of accessing care could be found in the linked routine data or available source data. Participant could not be contacted telephonically so we were unable to confirm their care status.*

**Table 3:** Characteristics of mothers enrolled, comparing those with and without any probable gaps in vertical transmission prevention steps. Presented as n (%) unless specified.

Characteristic	All women	No probable gaps	One probable gap	Two or more probable gaps	Any probable gaps	p-value (any vs no gaps)
Total number enrolled	336	203	93	40	133	
Number with enrolment interview data	330	202	90	38	128	
Median maternal age at delivery, years, (IQR)	32 (28–36)	33 (29–37)	31 (28–35)	29 (25–33)	31 (27–35)	0.002
< 25 years	40 (12)	18 (9)	12 (13)	10 (26)	22 (17)	0.013
≥ 25, < 35 years	193 (58)	115 (57)	57 (63)	21 (55)	78 (61)	
≥ 35 years	97 (29)	69 (34)	21 (23)	7 (18)	28 (22)	
Completed high school	95 (29)	60 (30)	26 (29)	9 (24)	35 (27)	0.645
Currently employed	95 (28)	55 (27)	34 (38)	6 (16)	40 (30)	0.553
Receiving a government grant	241 (73)	145 (72)	69 (77)	27 (71)	96 (75)	0.521
Poverty tertiles (asset score and employment)						
Lowest score (most poverty)	121 (37)	70 (35)	34 (38)	17 (45)	51 (40)	0.277
Middle	110 (33)	74 (37)	20 (22)	16 (42)	36 (28)	
Highest (least poverty)	99 (30)	58 (29)	36 (40)	5 (13)	41 (32)	
Married/cohabiting	157 (48)	99 (49)	44 (49)	14 (37)	58 (45)	0.512
First pregnancy	36 (11)	20 (10)	10 (11)	6 (15)	16 (12)	0.528
Planned pregnancy	71 (22)	43 (21)	22 (24)	6 (16)	28 (22)	0.899
Newly diagnosed with HIV in this pregnancy	45 (14)	28 (14)	13 (14)	4 (11)	17 (13)	0.881
Disclosed to anyone	318 (96)	195 (97)	87 (97)	36 (95)	123 (96)	0.835
Disclosed to partner	246 (73)	155 (79)	65 (70)	26 (65)	91 (74)	0.253
Current regimen						
Tenofovir, Efavirenz, Emtricitabine	128 (39)	78 (39)	47 (52)	25 (66)	50 (39)	0.887
Tenofovir, Lamivudine, Dolutegravir	184 (56)	112 (55)	38 (42)	12 (32)	72 (56)	
Other	18 (5)	12 (6)	5 (6)	1 (3)	6 (5)	
ART history						
Started ART in this pregnancy	50 (15)	32 (16)	13 (14)	5 (13)	18 (14)	< 0.001
ART-experienced, ≥ 1 previous interruption	111 (34)	48 (24)	39 (43)	24 (63)	63 (49)	
ART-experienced, reports no previous interruptions	169 (51)	122 (60)	38 (42)	9 (24)	47 (37)	
Duration on ART before delivery, years (n = 280 with ART experience)	6.0 (3.9–9.0)	6.1 (3.9–9.4)	6.8 (4.3–9.2)	4.3 (2.8–6.5)	5.8 (3.4–8.6)	0.429

Description of gaps in VTP steps

**Table 4:** Summary of 179 probable gaps (including confirmed and unconfirmed) in vertical transmission prevention steps

	Number of probable gaps	Mother was successfully traced (gap confirmed)	Reported reasons for gap^a^	Successfully linked to care following tracing
**All probable gaps**	179 gaps (133 MIPs)	100 (56%) of probable gaps	63 (63%) of traced mothers	47 (47%) of those traced
**Mother did not link to HIV care after delivery**	54 MIPs 30% of all gaps 16% of mothers	37 (69%) of probable gaps	22 (59%) of traced mothers provided reasons: mobility Eastern Cape (7), transfer documentation challenges (8), work (5), mobility local (4), fear of stigma or being scolded (4), mental health (1), side effects (1), “no time” (1), death in family (1), transport money (1)	10 (27%) of those traced
**Mother had gap of > 90 days with no ART dispensed after linking to care postpartum**	43 MIPs 24% of all gaps 13% of mothers	29 (67%) of probable gaps	21 (72%) of traced mothers provided reasons: mobility Eastern Cape (10), mobility local (6), transfer documentation challenges (5), fear of stigma or being scolded (3), work (2), alcohol use (1), tired of attending (1)	19 (66%) of those traced
**No infant 10-week HIV test**	26 MIPs 14% of all gaps 10% of infants	12 (46%) of probable gaps	7 (58%) of traced mothers provided reasons: mobility Eastern Cape (4), mobility Gauteng (1), transfer documentation challenges (1), tired of attending (1)	10 (83%) of those traced
**No infant 6-month HIV test**	56 MIPs 32% of all gaps 17% of infants	22 (39%) of probable gaps	13 (59%) of traced mothers provided reasons: mobility Eastern Cape (13), negative 10-week result (1)	8 (36%) of those traced

a number of women reporting the reason in brackets; multiple reasons could be reported per gap

**Table 5:** Overlap of mother and infant gaps in care. Presented as n (%) out of 335 MIPs at 10 weeks and 332 MIPs at six months postpartum. (Numbers exclude infant deaths: one death before 10 weeks and three deaths between 10 weeks and 6 months postpartum)

	Mother linked to care postpartum	Mother did not link to care postpartum
Infant 10-week HIV test done	271 (81)	53 (16)
No infant 10-week HIV test	10 (3)	1 (< 1)
	Mother in care around 6 months	Mother not in care around 6 months
Infant 6-month HIV test done	223 (67)	41 (12)
No infant 6-month HIV test	53 (16)	15 (5)

## Abbreviations

ART: antiretroviral therapy
D2C: Data to Care
LMIC: low- and middle-income countries
MIP: mother-infant pair
MOU: midwife obstetric unit
PHDC: Provincial Health Data Centre
WC: Western Cape
